# Assessment of cranial reconstruction utilizing various implant materials: finite element study

**DOI:** 10.1007/s10856-024-06816-9

**Published:** 2024-08-13

**Authors:** Yomna H. Shash

**Affiliations:** Biomedical Engineering Department, Faculty of Engineering, Helwan University, Cairo, Egypt

**Keywords:** Craniectomy, Cranioplasty, Cranial implant, Hydroxyapatite, PMMA

## Abstract

The human head can sometimes experience impact loads that result in skull fractures or other injuries, leading to the need for a craniectomy. Cranioplasty is a procedure that involves replacing the removed portion with either autologous bone or alloplastic material. While titanium has traditionally been the preferred material for cranial implants due to its excellent properties and biocompatibility, its limitations have prompted the search for alternative materials. This research aimed to explore alternative materials to titanium for cranial implants in order to address the limitations of titanium implants and improve the performance of the cranioplasty process. A 3D model of a defective skull was reconstructed with a cranial implant, and the implant was simulated using various stiff and soft materials (such as alumina, zirconia, hydroxyapatite, zirconia-reinforced PMMA, and PMMA) as alternatives to titanium under 2000N impact forces. Alumina and zirconia implants were found to reduce stresses and strains on the skull and brain compared to titanium implants. However, PMMA implants showed potential for causing skull damage under current loading conditions. Additionally, PMMA and hydroxyapatite implants were prone to fracture. Despite these findings, none of the implants exceeded the limits for tensile and compressive stresses and strains on the brain. Zirconia-reinforced PMMA implants were also shown to reduce stresses and strains on the skull and brain compared to PMMA implants. Alumina and zirconia show promise as alternatives to titanium for the production of cranial implants. The use of alternative implant materials to titanium has the potential to enhance the success of cranial reconstruction by overcoming the limitations associated with titanium implants.

**Graphical Abstract:**

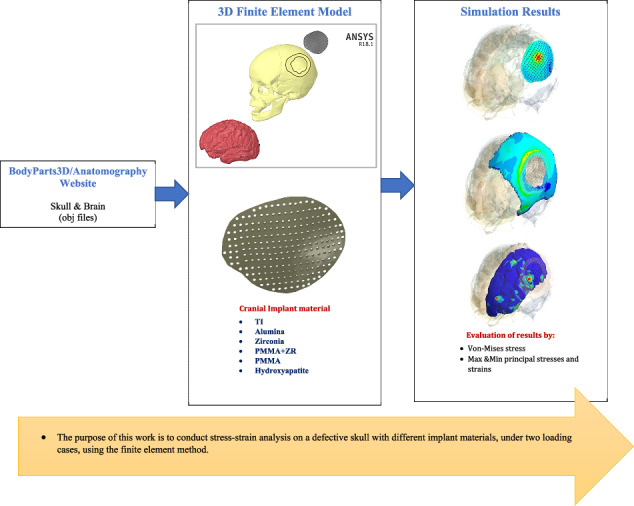

## Introduction

Human heads are often impacted during car crashes, falls, or sports-related events, leading to one of the main causes of accidental death: mechanically induced head injuries. A head injury encompasses a wide range of injuries to the scalp, skull, brain, underlying tissue, and blood vessels, and can be categorized into scalp injury, skull fracture, brain injury, or a combination of these [1].

Craniectomy is a surgical procedure involving the removal of a portion of the skull. Injuries can cause swelling and inflammation, increasing brain fluid and intracranial pressure, potentially damaging brain tissue and leading to death, coma, or irreversible brain damage [2]. To alleviate this pressure and prevent complications, craniectomy is performed. Cranioplasty, on the other hand, is the surgical correction of a cranial bone defect resulting from an injury or craniectomy, aiming to restore the cranial vault’s morphology and function, improve skull appearance, preserve the brain, and prevent neurological issues or alterations in cerebrospinal fluid [3].

The materials used in cranioplasty can be biological or synthetic, with options including autografting (using the patient’s own tissue), allografting (using a donor’s tissue), or alloplastic materials like metals, ceramics, and polymers [4]. The choice of implant material is crucial for successful cranial reconstruction, as it affects the distribution of stresses and strains on the implant, skull, and brain [5, 6]. Some researchers recommend stiff materials such as ceramics and metals for their durability and quality, hypothesizing that they transfer less stress to surrounding areas [7–9]. Others suggest using soft implants like polymers to dampen stresses and protect the brain [10, 11].

Titanium is the commonly used material for cranial reconstruction in the fabrication of cranial implants because of its rigidity, strength, biocompatibility, and good corrosion resistance [6]. However, titanium has drawbacks, including casting issues, the requirement of surface modification, and incompatibility with imaging techniques such as CT and MRI, as it produces scatter artifacts that interfere with radiologic interpretation [12]. Recent studies have clarified that titanium has a toxic effect, as its particles and ions are deposited into surrounding tissues due to the corrosion and wear of implants [13, 14]. This can lead to bone loss due to inflammatory reactions and osseointegration failure of the implant [14]. In addition, titanium implants can induce hypersensitive reactions such as erythema, urticaria, eczema, swelling, pain, and necrosis [15, 16]. They have also been implicated in many clinical problems, such as surface degradation and contamination related to peri-implantitis [16]. These drawbacks have prompted researchers to find alternative materials for cranial implants.

Several crucial requirements must be met when choosing cranial implants, including biocompatibility and adequate mechanical properties to tolerate function-related stress [5]. Other important factors must be taken into account in the selection of implant material, such as low cost, ease of manufacture, short preparation time, and technical readiness for clinical application.

One viable option for cranial reconstruction is to choose a cranial implant from stiff bioceramic materials such as alumina or zirconia instead of titanium. These ceramics are characterized by having good properties such as good mechanical properties (durable in compression), good thermal and chemical properties (low thermal conductivity and excellent corrosion resistance), biocompatibility, durability, and excellent esthetics [17, 18]. Bioceramics are also strong, nontoxic, chemically stable within the biological environment, and do not induce inflammatory reactions [19, 20]. Consequently, bioceramic materials can provide functional and long-lasting cranial implants. However, their effects on bone and the underlying brain tissues are unclear, and further research is required in the near future.

Calcium hydroxyapatite is a natural component of teeth and bone. Synthetic hydroxyapatite (HA) is used as an active soft bioceramic material for teeth and bone replacements, as it can chemically bond to bone and promote bone growth on its surfaces [4, 21]. Additionally, it is biocompatible, does not have negative interactions with the body, and does not readily degrade or disappear due to its stability and low solubility [22]. Over the past few decades, hydroxyapatite cranioplasty has become increasingly popular. The unique biological properties of this material make it particularly suitable for patients undergoing decompressive craniectomy, where the primary goal is bone reintegration [21]. However, this material is not as strong as alumina or zirconia ceramics.

Acrylic, specifically methyl methacrylate, has recently been used in cranioplasty for the production of cranial implants. PMMA is a transparent and soft thermoplastic derived from the monomer methyl methacrylate [23]. The main advantage of PMMA over other metallic and ceramic materials is its cost-effectiveness, ease of production, strength, lightness, malleability when heated, ease of cleaning and maintenance, availability in cement form, and ability to be molded into various shapes [24]. Additionally, it exhibits good biocompatibility and physical and esthetic properties. However, PMMA is a soft material with a low modulus of elasticity compared to metallic and ceramic materials, and may fail under high or sudden forces [24].

Numerous efforts have been made to chemically modify PMMA by incorporating different reinforcing materials, such as metallic fillers, ceramic fillers, glass fibers, or carbon fibers, to enhance its mechanical properties. The addition of zirconia as a filler into PMMA significantly improves its mechanical properties [25]. The even size and distribution of the reinforcement contribute to increased strength, toughness, stability, and resistance to aging and thermal shock [26].

A computer-aided engineering tool called the finite element method (FEM) is used to examine how a design performs in real-world scenarios and solve biomedical problems [27, 28]. It is effective in studying biomechanical properties and predicting biomechanical responses under different mechanical conditions. FEM is particularly useful in maxillofacial surgeries and bone reconstruction processes as it aids in surgical planning, designing implants and instruments, and creating anatomically accurate prostheses. In cranial construction, for evaluating cranial implant materials, the finite element method can accurately reconstruct complex geometries, modify defects, propose different designs, simulate different materials under different conditions, and extract internal stresses and strains at any point [29, 30].

This research aimed to develop the cranioplasty process by using different materials in the production of cranial implants instead of titanium and then studying their mechanical performances using the FEM. Stress and strain analyses on the skull and brain were conducted after cranial reconstruction, employing different stiff and soft implant materials (alumina, zirconia, hydroxyapatite, zirconia-reinforced PMMA, and PMMA) under two different loading cases. The von Mises stresses, maximum and minimum principal stresses, strains, and total deformations were extracted for the skull and brain for evaluation. The null hypothesis predicted that the stiff implants (alumina, zirconia, and zirconia-reinforced PMMA) would exhibit the lowest stresses and strains on the skull and brain, representing the best scenario, especially under great or sudden forces, in contrast to the soft implants (PMMA and hydroxyapatite).

## Material and methods

### Model generation

The 3D geometry of the skull and brain was downloaded as OBJ files from the website “BodyParts3D/Anatomography” (BodyParts3D, Life Sciences Integrated Database Center, Japan) [31]. Using the software programs Solid Works and Space Claim, the models were converted to solid, modified, and repaired. The repair process included fixing curves, gaps, missing faces, splits, and extra edges, as well as merging very small faces. The skull had a large defect in the left parietal bone with an area of 11803.78 mm^2^.

**Fig. 1:**
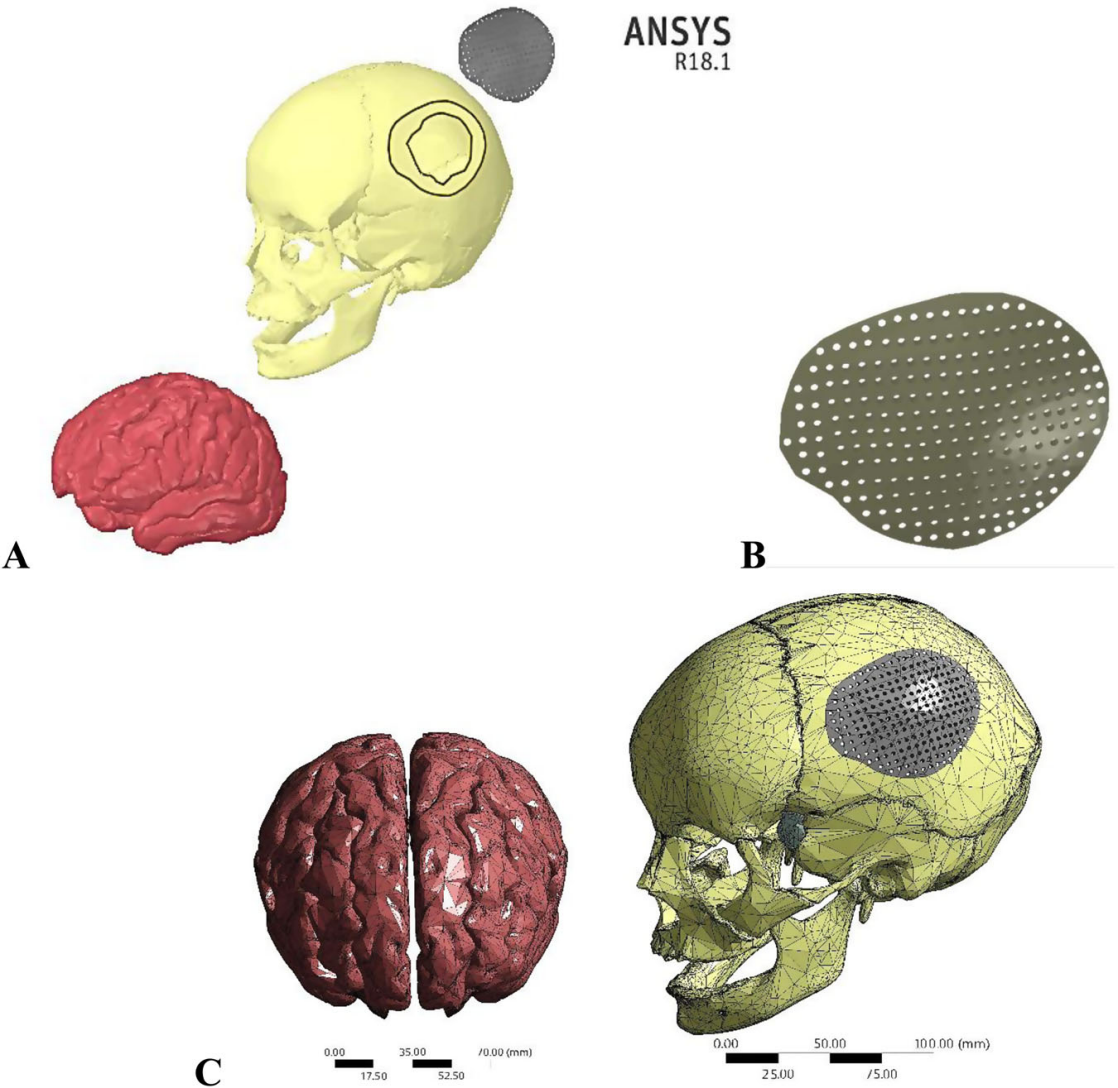
**A** Brain, defective skull and implant, **B** Implant design, and **C** Meshing

To design the cranial implant, the patient’s skull was assumed to be symmetrical along its mid-sagittal plane. Therefore, the missing bone fragment in the left parietal bone was mirrored from the right parietal bone to create a realistic situation. The implant had an irregular shape to fit the defective portion, with multiple holes of 2 mm in diameter based on previous studies [29, 32]. Once the previous steps of creating the defective skull and the implant were completed, all parts (skull, implant, and brain) were assembled and exported to ANSYS software (ANSYS 18.1, Houston, TX, USA) as shown in Fig. 1A, B. In ANSYS, all bodies were considered to be fully connected via their contact surfaces, with no relative movement (fully bonded). Additionally, the skull-implant contact area was assumed to be fully osteointegrated.

### Material selection

In this section, all materials were assumed to be homogeneous, isotropic, and linearly elastic in an effort to simplify the complexity and reduce computational time [33]. These assumptions are commonly used when analyzing the deformation of a solid body, determining the relationship between applied forces and resulting shape changes, and understanding the relationship between stresses and strains in the material [34].

In this study, the cranial implant was simulated using a variety of materials including traditional materials (titanium), stiff ceramics (alumina and zirconia), soft ceramic (hydroxyapatite), composite (zirconia reinforced PMMA), and soft polymer (Poly methyl methacrylate PMMA). The properties of the skull, brain, and implants are outlined in Table 1.

**Table 1:** The properties of the skull, brain, and implant

	Elastic Modulus (MPa)	Poisson Ratio	Density (kg/m^3^)	Tensile Strength (MPa)	Compressive Strength (MPa)	Ref.
Skull	15,000	0.3	1800	140	180	[32]
Brain	0.0015	0.45	1040	1e-3	50e-3	[47, 48]
Titanium Alloy	110,000	0.34	4500	862	848	[32]
Alumina	380,000	0.45	3950	275	2600	[43]
Zirconia	210,000	0.45	6000	500	2500	[49]
Hydroxyapatite	7000	0.27	1656	48	450	[50]
PMMA(80%)+ Zirconia(20%)	42,560	0.35	2080	160	594	[26, 51]
PMMA	5000	0.37	1185	71	120	[44]

### Meshing adjustment

The ANSYS software was utilized to create a three-dimensional mesh using an “adaptive” function and fine elements, as depicted in Fig. 1C. The minimum element size of the mesh for all models was 0.2 mm, based on the convergence test. The number of nodes and elements is presented in Table 2.

**Table 2:** The number of elements and nodes

	No. of Elements	No. of Nodes
Defected Left Parietal Bone	73,643	130,940
Right Parietal Bone	76,234	134,947
Frontal Bone	87,024	152,476
Brain	706,287	1,1119,773
Implant	29,964	56,722

### Loading and constraint conditions

**Fig. 2:**
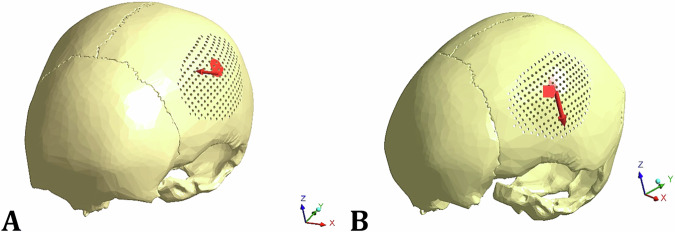
Two cases of loading: **A** Force.1, and **B** Force.2

The loading thresholds for clinically significant skull fractures were ~2450 N for men and 2000 N for women, according to references [6, 35]. Additionally, a force of nearly 2000 N was required to fracture the parietal bone [35]. Therefore, in this study, a uniformly distributed force of 2000 N was applied in two different directions (Case 1 and Case 2), as shown in Fig. 2. In Case 1, the force was in the negative direction of the x-axis, while in Case 2, the force was in the negative direction of the z-axis.

**Fig. 3:**
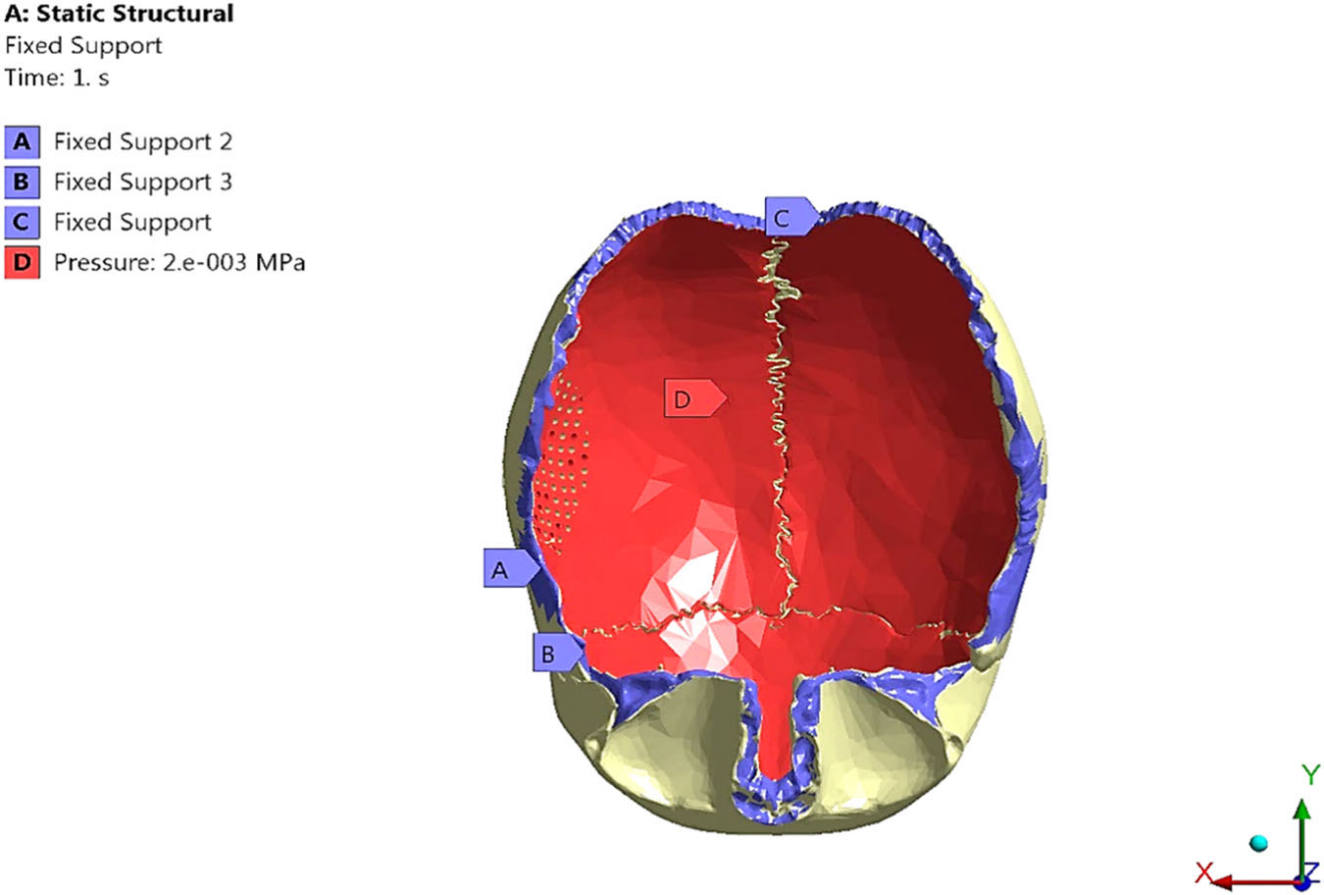
Fixed supports and intracranial pressure

Furthermore, a static pressure of 2000 Pascal was applied (Fig. 3) on the inner surface of the skull and evenly distributed over an implant area to simulate intracranial pressure conditions [6]. As for the boundary conditions, the nodes of the inferior border of parietal bones were constrained in all directions to prevent the displacement of the model under the force effect.

### Results evaluation

Analyses were performed using ANSYS software to extract the von Mises stresses, the maximum and minimum principal stresses and strains, and the total deformations to evaluate the results. The von Mises stresses were extracted to determine the maximum stresses generated on all parts (implants, skull, and brain). For implants and biological tissues (skull and brain), due to their different properties (ductile or brittle), the maximum (tensile) and minimum (compressive) principal stresses were extracted and compared to the tensile and compressive strengths to evaluate their responses (endurance or failure) [36, 37].

Moreover, the maximum and minimum principal strains were extracted for the skull and brain and compared with the permissible limits. Concentration and distribution of excessive strains may cause micro-damage if they exceed the thresholds. Damage to the skull occurred when the strain exceeded the thresholds of 2500–3000 με in tension and 5000–6000 με in compression [38]. For the brain, functional damage was produced when the strain exceeded the range of 100,000–200,000 με in tension and compression [29]. Additionally, in this research, the total deformations of the skull and brain were investigated.

## Results

### Max von Mises stresses

In this study, five different materials (zirconia, titanium, zirconia-reinforced PMMA, hydroxyapatite, and PMMA) were utilized. The maximum von Mises stresses generated on the implant, skull, and brain were extracted and presented in Table 3 under two different forces.

**Table 3:** Max von Mises stresses (MPa) on all parts, by using different implant materials, under two different forces

		*Al*	*ZR*	*TI*	*PMMA+ZR*	*HA*	*PMMA*
Force.1	Cranial Implant	236.31	232.48	225.73	210.72	193.14	193
	Skull	40.723	40.87	41.18	42.23	52.07	112.45
	Brain	4.62e-6	5.3 e-6	6.52 e-6	1.06 e-5	2.04 e-5	3.86 e-5
Force.2	Cranial Implant	163.73	162.66	161.58	158.16	152.9	150.1
	Skull	43.043	43.37	43.50	45.165	47.48	54.54
	Brain	1.95e-6	2.24e-6	2.60e-6	4.437e-6	8e-6	2.52e-5

Under force 1, the maximum von Mises stress on the cranial titanium implant was 225.73 MPa. This value increased by 4.6% and 2.9% on alumina and zirconia implants, and decreased by 6.6%, 14.4%, and 14.5% on PMMA+ZR, hydroxyapatite, and PMMA implants, respectively. Consequently, the maximum von Mises stress on the skull decreased by 1.1% and 0.75% when using alumina and zirconia implants, and increased by 5.49% and 26.4% when using PMMA+ZR and hydroxyapatite implants, compared to the titanium implant. The use of a PMMA implant led to a significant increase in stress on the skull to 112.45 MPa. On the brain, the maximum von Mises stress was 6.52e-6 MPa when utilizing a titanium implant. This value decreased by 29.1% and 18.7% when using alumina and zirconia implants, respectively, but increased by 61.04% when using a PMMA+ZR implant compared to the titanium implant. Hydroxyapatite and PMMA had notable effects on the brain, increasing its stresses to 2.04e-5 and 3.86e-5 MPa, respectively.

Under force 2, the maximum von Mises stress on the cranial titanium implant was 161.58 MPa. This stress value showed slight changes in alumina and zirconia implants. The use of an alumina implant resulted in a 1.05% decrease in stress on the skull and a 25% decrease in stress on the brain compared to a titanium implant. With a zirconia implant, the stresses on the skull and brain decreased by 0.29% and 13.8%, respectively. When using PMMA+ZR, the stresses on the implant, skull, and brain changed by −2.11%, 3.8%, and 70.6%, respectively, compared to the titanium implant. Hydroxyapatite and PMMA implants led to increases of 9.1% and 25.3% in skull stresses, respectively, compared to titanium implants. Additionally, the stress on the brain significantly increased to 8e-6 and 2.52e-5 MPa.

**Fig. 4:**
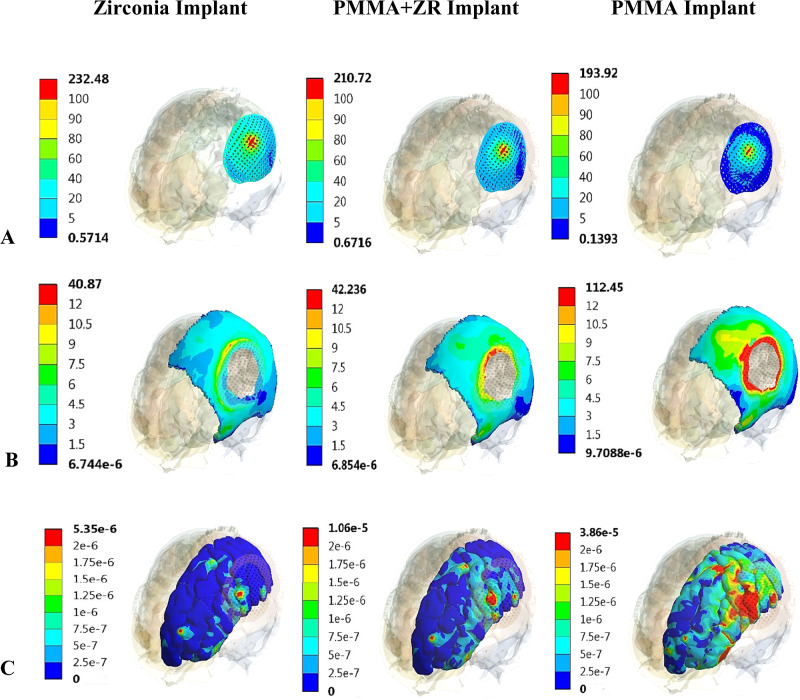
Distribution of von-Mises stresses(MPa) on: **A** Implant, **B** Defective skull, and **C** Right portion of the brain, under force.1, using zirconia, PMMA+ZR and PMMA implants

Figure 4 illustrates the distribution of von Mises stresses (MPa) on the implant, defective skull, and right portion of the brain, under force.1, using Zirconia, PMMA+ZR and PMMA implants.

### Peak Max and Min principal stresses

The maximum (tensile) and minimum (compressive) principal stresses were extracted in Table 4 for the cranial implant, skull, and brain, and compared to the limits according to failure theories [36, 37] in order to investigate the yielding/failure behavior of each part.

**Table 4:** Max and Min principal stresses (MPa) on implant, skull and brain, by using different implant materials, under two different forces

			*Al*	*ZR*	*TI*	*PMMA+ZR*	*HA*	*PMMA*
Force.1	Cranial Implant	*Max*	170.54	157.51	156.23	143.99	125.07	120.95
		*Min*	−235.19	−229.74	−221.49	−206.73	−191.49	−132.4
	Skull	*Max*	25.5	25.95	26.43	27.23	36.11	40.08
		*Min*	−29	−29.05	−30.22	−33.47	−41.32	−101.2
	Brain	*Max*	9.33e-6	1.08 e-5	1.33 e-5	2.22 e-5	4.47 e-5	7.63 e-5
		*Min*	−9.47e-6	−1.20 e-5	−1.55e-5	−2.51 e-5	−5.30e-5	−9.51 e-5
Force.2	Cranial Implant	*Max*	98.435	95.654	89.162	88.823	84.5	80.58
		*Min*	−190.08	−188.37	−167.25	−163.48	−157.5	−150.8
	Skull	*Max*	43.55	43.84	43.99	45.586	51.23	57.453
		*Min*	−47.817	−58.15	−63.11	−67.101	−73.85	−88.25
	Brain	*Max*	2.87e-6	3.44e-6	4.8e-6	8.69e-6	2.03e-5	5.45e-5
		*Min*	−1.98e-6	−2.82e-6	−4.01e-6	−7.79e-6	−2.19e-5	−4.97e-5

In force 1, on the cranial implant, the peak max principal stress increased by 9.1% and 0.81% on alumina and zirconia implants, and decreased by 7.8%, 19.9%, and 22.5% on PMMA+ZR, hydroxyapatite, and PMMA implants compared to the titanium implant. Additionally, the peak min principal stress increased by 6.18% and 3.72% on alumina and zirconia implants, and decreased by 6.6%, 13.5%, and 40.2% on PMMA+ZR, hydroxyapatite, and PMMA implants. For the skull, the peak max and min principal stresses decreased by (3.6% and 4%) and (1.81% and 3.8%) using alumina and zirconia implants, and increased by (3.02% and 10.75%) and (36.6% and 36.73%) using PMMA+ZR and hydroxyapatite implants. Using a PMMA implant, the peak max and min principal stresses increased to (40.08 MPa and −101.2 MPa) respectively on the skull. Regarding the brain, the use of hydroxyapatite and PMMA implants greatly increased the peak max and min principal stresses to (4.47 e-5 MPa and −5.30 e-5 MPa) and (7.63 e-5 MPa and −9.51 e-5 MPa) respectively.

In force 2, the peak max and min principal stresses on the titanium implant were (89.16 MPa and −167.25 MPa). The max and min principal stresses increased by (10.4% and 14%) and (7.2% and 12.6%) on alumina and zirconia implants, and decreased by (0.38% and 2.25%), (5.2% and 5.8%), and (9.6% and 9.8%) on PMMA+ZR, hydroxyapatite, and PMMA implants respectively. On the skull, the max and min principal stresses decreased by (1% and 24%) and (0.34% and 7.8%) using alumina and zirconia implants, and increased by (3.62% and 6.32%), (16.4% and 17%), and (30.6% and 39%) using PMMA+ZR, hydroxyapatite, and PMMA implants respectively. Consequently, compared to titanium implants, the max and min principal stresses on the brain decreased using alumina and zirconia implants, and greatly increased using other materials.

**Fig. 5:**
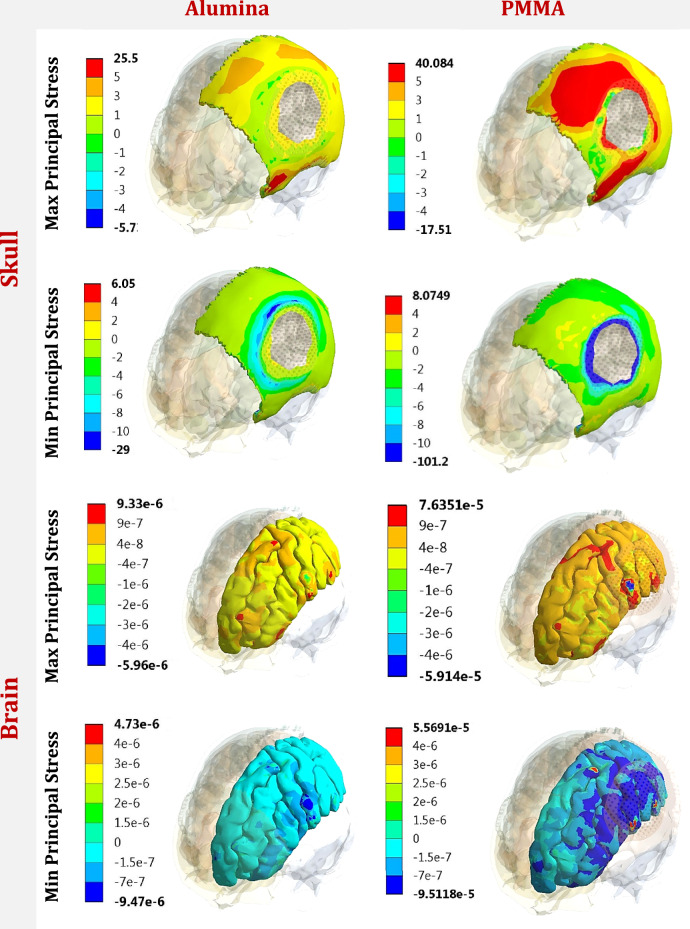
Distribution of maximum and minimum principal stresses (MPa) on skull and brain, under force.1, using alumina and PMMA implants

**Fig. 6:**
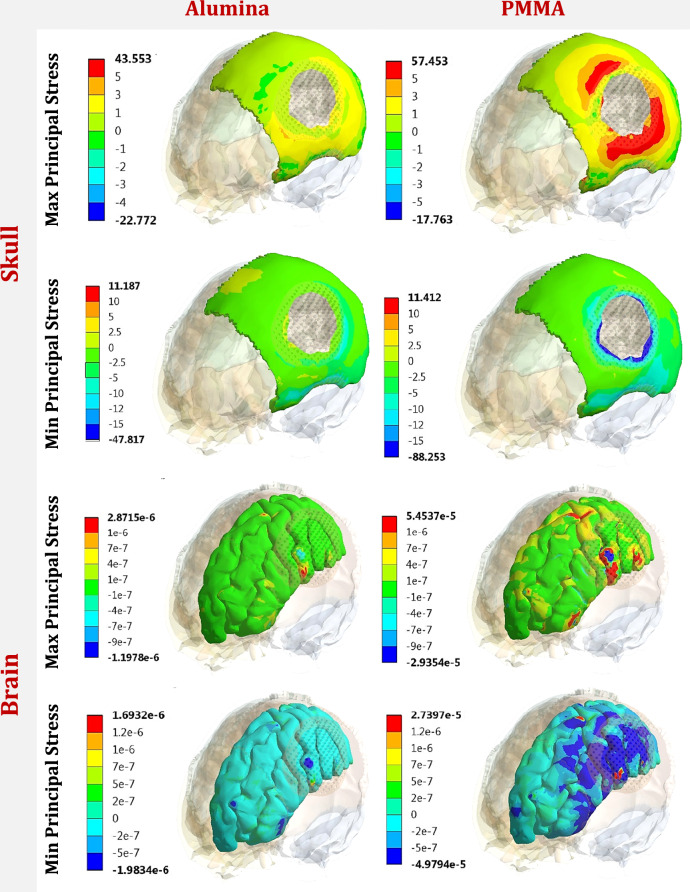
Distribution of maximum and minimum principal stresses (MPa) on skull and brain, under force.2, using alumina and PMMA implants

**Fig. 7:**
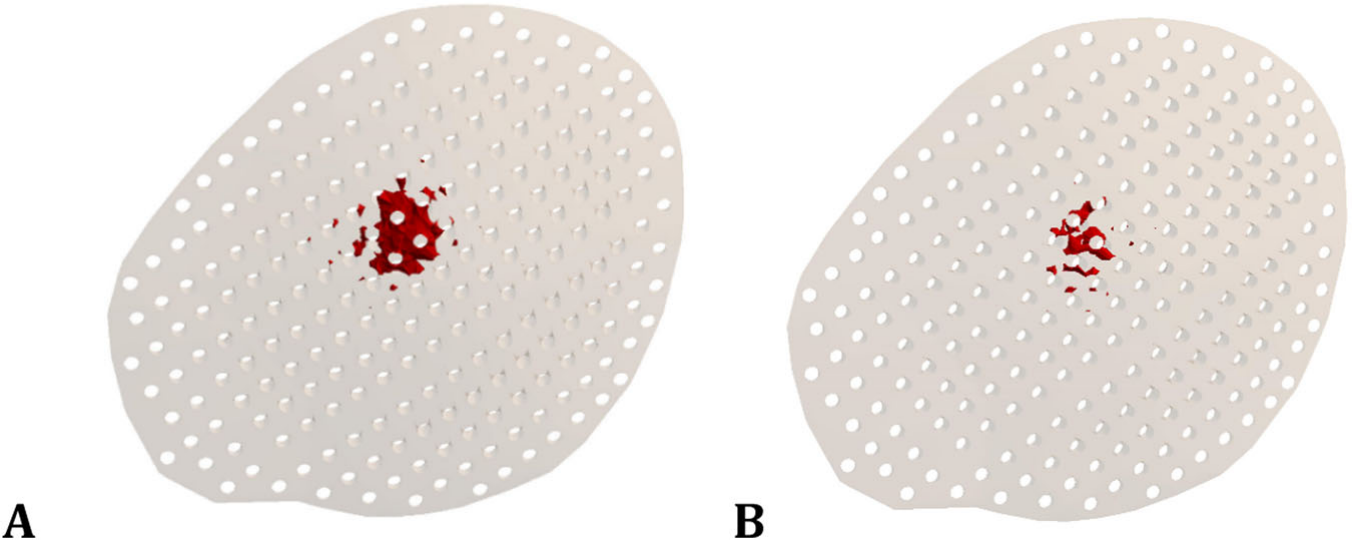
Expected fracture zones in PMMA implants, under: **A** Force.1 and **B** Force.2

Figures 5 and 6 illustrate the distribution of maximum and minimum principal stresses (MPa) on the skull and brain under force 1 and force 2 using alumina and PMMA implants. Figure 7 illustrates the zones (red) in which the tensile stresses on the PMMA implant exceeded the allowable tensile limit of 71 MPa (Table 1) under force 1 and force 2, respectively. These zones might be subjected to fracture under the assumed loading conditions.

### Peak Max and Min principal strains

For the skull and brain, the maximum and minimum principal strains were computed in Table 5, as microstrains can cause damage if they exceed critical limits. In force 1, using a titanium implant, the maximum and minimum principal strains were (1961 and −2159 με) and (4061 and −3816 με) on the skull and brain, respectively. An alumina implant decreased the maximum and minimum principal strains by (1.9 and 0.34%) and (25.9 and 39.57%) on the skull and brain, respectively. By using a zirconia implant, the maximum and minimum principal strains were decreased by (1.2 and 0.34%) and (14.3 and 23.48%) on the skull and brain, respectively. Using PMMA+ZR, the maximum and minimum principal strains were increased by (3.9 and 1.34%) and (43.9 and 61.8%) on the skull and brain. A PMMA implant increased the maximum and minimum principal strains to (3591 and −7146 με) and (19411 and −25447 με) on the skull and brain.

**Table 5:** Max and Min principal strain (με) on skull and brain, by using different implant materials, under two different forces

			*Al*	*ZR*	*TI*	*PMMA+ZR*	*HA*	*PMMA*
Force.1	Skull	*Max*	1923	1937	1961	2038	2416	3591
		*Min*	−2152	−2152	−2159	−2188	−2960	−7146
	Brain	*Max*	3006	3480	4061	5847	11,286	19,411
		*Min*	−2306	−2920	−3816	−6177	−13,095	−25,447
Force.2	Skull	*Max*	2395	2409	2415	2440	2659	2952
		*Min*	−3523	−3545	−3588	−3607	−3652	−3890
	Brain	*Max*	1309	1489	1720	2326	5855	15,349
		*Min*	−798	−1003	−1479	−2945	−7668	−16,619

In force 2, using an alumina implant, the maximum and minimum principal strains were decreased by (0.82 and 1.81%) and (23.89 and 46.04%) on the skull and bone compared to a titanium implant. By using PMMA+ZR, the maximum and minimum principal strains were increased by (7.2 and 0.52%) and (35.2 and 99.1%) on the skull and bone, respectively. A PMMA implant increased the maximum and minimum principal strains to (2952 and −3890 με) and (15,349 and −16,619 με) on the skull and brain.

**Fig. 8:**
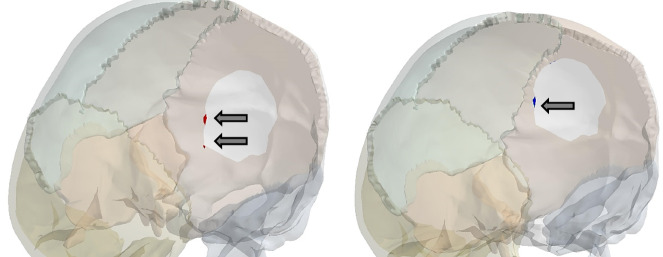
Expected fracture zones in the skull, under tension (red) and compression(blue), using PMMA implant, under force.1

Figure 8 illustrates the zones in which the tensile and compressive strains on the skull exceeded the allowable tensile (3000 με) and compressive (6000 με) limits respectively, using the PMMA implant under force 1. These zones of the skull were expected to be fractured.

### Total deformation

**Fig. 9:**
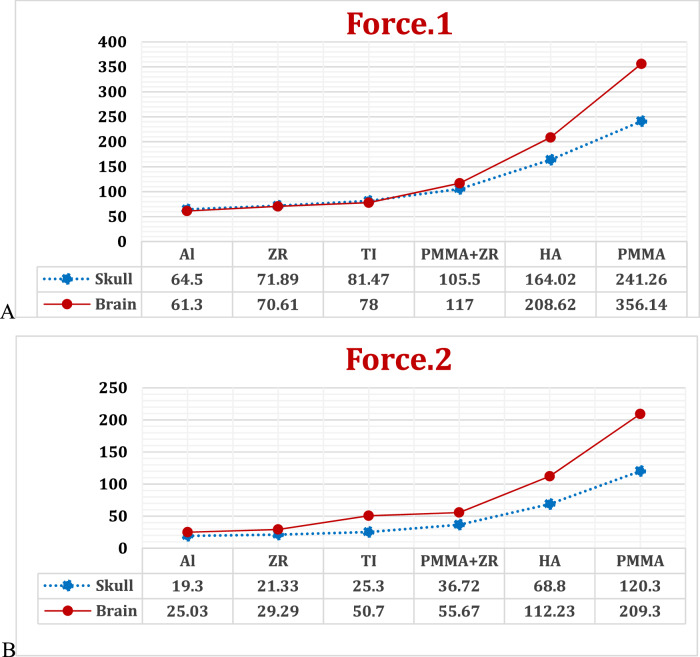
Max deformation (μm) on skull and brain, by using different implant materials, under: **A** Force.1 and **B** Force.2

The maximum deformations of the skull and brain were calculated (Fig. 9) to determine the changes that occurred in their shape or size as a result of the applied forces.

Under force 1 and force 2, on the skull, the maximum deformation values were 81.47 and 25.3 μm when using a titanium implant. These values decreased by 20.8% and 23.71%, and 11.75% and 15.6% when using alumina and zirconia implants, respectively. However, the deformation values increased by 29.4% and 45.1% when using PMMA+ZR under force 1 and force 2, respectively. By using a hydroxyapatite implant, the deformation values increased to 164.02 μm and 68.8 μm under the two forces. The values significantly increased to 241.26 μm and 120.3 μm when using a PMMA implant.

Using alumina and zirconia implants decreased the deformation of the brain by 21.4% and 50.63%, and 9.47% and 42% under the two cases, respectively. In contrast, the PMMA+ZR implant increased the deformation of the brain by 50% and 9.8% under the two forces. The hydroxyapatite implant increased the brain deformation to 208.62 μm and 112.23 μm, respectively, while the PMMA implant increased the deformation to 356.14 μm and 209.3 μm under the two forces, respectively.

## Discussion

A neurosurgical technique called cranioplasty is used to correct or reshape abnormalities or flaws in the skull. Cranioplasty is often recommended to treat birth defects or as a result of post-traumatic injuries. In addition to restoring the continuity and integrity of the skull, as well as its previous appearance, cranioplasty can also stabilize intracranial pressure and create a stable intracranial state. Furthermore, it promotes brain metabolism, restores cranial nerve function, reestablishes the protective layer around the brain, and reduces the negative effects of the defect [1–3].

The materials and designs used in cranioplasty are crucial. Wan et al. [30] compared the stress distribution, maximum stress, strain distribution, and maximum deflection of four different implants and their corresponding defective skulls. None of the four implants or defective skulls broke down or deformed under a 500 N load. Currently, titanium alloy (Ti-6Al-4V) is the most commonly used material for cranial implants due to its high biocompatibility, durability, strength, corrosion resistance, and lightweight nature. However, one of the main drawbacks of titanium is its allergenic potential [13, 14]. Other issues include problems with casting, the need for surface modification, esthetic concerns, and incompatibility with both CT and MRI imaging techniques [12]. These challenges with titanium have led manufacturers to seek alternative materials for use in cranioplasty.

Hydroxyapatite (HA), from the calcium phosphate family, has been widely used as a bone substitute in various medical specialties, including dentistry, and more recently in neurosurgery. Custom-made cranial implants made of HA have become increasingly popular in recent years [4, 21, 22]. These implants are constructed using a 3D CT scan provided by the attending neurosurgeon to the manufacturer. The company then creates a prototype, which must be reviewed and approved by the neurosurgeon before the final implant is produced. The use of HA has grown due to its well-known osseointegration properties and minimal risk of postoperative infection. Additionally, hydroxyapatite is considered a bone scaffold, particularly in children, to support the growth and remodeling of new cranial bone. Clinical studies have shown promising results with hydroxyapatite implants, especially in the pediatric population, demonstrating encouraging outcomes in terms of biocompatibility and osteoconductivity that promote osseous bridging at the interface between the prosthesis and skull [39].

In the field of biomedical applications, alumina and zirconia bioceramics are commonly used in orthopedics and dentistry for applications such as knee prostheses, maxillofacial reconstruction, and as bone substitutes in dental implants [5, 40, 41]. These ceramics have gained attention in neurosurgery due to their mechanical resistance and bone-like appearance [42]. Alumina has shown superior behavior compared to titanium in terms of mechanical properties and compatibility with bone tissue [43].

Polymeric materials are utilized as biocomposite matrices or soft tissue replacements in various medical applications. In neurosurgery, porous ethylene, polyether ketone (PEEK), and poly methyl methacrylate (PMMA) are commonly used polymers [23, 24]. PMMA, a polymerized form of methyl methacrylate, solidifies during polymerization and is used in surgical procedures due to its transparency, ease of preparation, mechanical qualities, and affordability [23, 44]. PMMA is well-tolerated by the body and is used as cement or pre-shaped solid implants in neurosurgery for procedures such as cranioplasty and vertebral stabilizations. Studies have shown that PEEK and PMMA implants outperform autologous bone implants and may be preferred over titanium implants. Jindal et.al [45] used finite element analysis to compare the various cranial implant materials (autologous bone, PMMA, PEEK, and Ti-6Al-4V) fitted to a defective skull. According to the findings, PEEK and PMMA implants outperform autologous bone implants and may be preferred over titanium implants.

PMMA is softer than metallic and ceramic materials due to its lower modulus of elasticity, making it more susceptible to failure under strong or abrupt forces [23]. The addition of zirconia particles has been shown to improve the flexural strength of PMMA, with the concentration of zirconia impacting the mechanical properties and enhancing the clinical performance of dental and medical prostheses [24, 25].

The present study aimed to conduct stress and strain analysis on the skull and brain after cranial reconstruction using five different implant materials (alumina, zirconia, hydroxyapatite, zirconia-reinforced PMMA, and PMMA) as alternatives to titanium under two loading cases. The von Mises stresses, maximum and minimum principal stresses and strains, and total deformations were extracted for the skull and brain for result evaluation. The null hypothesis, assuming that stiff implants (alumina, zirconia, and zirconia-reinforced PMMA) would exhibit the lowest stresses and strains on the skull and brain, representing the best scenario compared to soft implants (PMMA and hydroxyapatite), was partially accepted. The results showed that the stresses transferred to bone tissues from different implants varied due to their mechanical properties. Stiff ceramic implants (alumina and zirconia) decreased stresses and deformations on the skull and brain but increased stresses on themselves. Conversely, soft implants (hydroxyapatite and PMMA) increased stresses on the skull and brain. Zirconia-reinforced PMMA implants decreased stresses and strains on the skull and brain compared to PMMA implants.

In the failure study of prosthetic parts (implants), the maximum and minimum principal stresses were extracted and compared with the tensile and compressive yield strengths under the two loading cases. For titanium, alumina, zirconia, and PMMA+ZR implants, the peak values of maximum principal stresses did not exceed their tensile yield strengths, nor did the peak values of minimum principal stresses surpass the compressive yield strengths, suggesting no deformation or damage under the loading conditions. However, for hydroxyapatite and PMMA implants, stress values exceeded limits and could potentially fail under a 2000 N force.

For the skull bone, the maximum (tensile) and minimum (compressive) principal stresses were computed (Table 4) and compared to the tensile and compressive yield strengths (Table 1). Although PMMA+ZR, HA, and PMMA implants significantly increased the stresses on the skull compared to titanium implants, the values did not exceed the permissible limits. Additionally, the maximum and minimum strains were calculated and compared to the physiological limits. Excessive strain can damage the implant-bone interfaces and alter the microstructure of bone. For the skull bone, a microstrain level over 5000–6000 was commonly indexed as the failure threshold under compression and 2500–3000 under tension [38]. The results (Table 5) demonstrated that the level of microstrain using PMMA implants exceeded the critical thresholds under force.

For the brain, the maximum and minimum principal stresses were within the permissible limits of 1 kPa under tension and 50 kPa under compression [46]. Besides, the maximum and minimum principal strains did not exceed the limits. These limits were 100,000, 200,000, and 250,000 με corresponding to reversible damage, functional damage, and structural damage [29].

The limitations of this study included that the materials were considered to be isotropic and linearly elastic. It is important to consider this limitation when interpreting the results, as the brain tissue and the skull are more complex and anisotropic. Other limitations were related to the construction of the implant, as changing the design or location may change the results. In addition, the determination of load and boundary conditions remained hypothetical and might not fully reflect the real clinical situation. Hence, further research and many controlled clinical trials on cranial implants are required in the near future.

## Conclusion

Cranioplasty is performed to improve the shape and function of the skull and protect the brain from neurological issues and changes in fluid. There are two types of materials used in cranioplasty: synthetic and biological. The success of cranial reconstruction depends on the choice of implant material. Some researchers suggest using stiff materials for durability, while others recommend soft materials to reduce stress on surrounding areas. Titanium has been the preferred material for cranial implants due to its compatibility and durability, but drawbacks have led to the search for alternative materials.

This study aimed to enhance cranial reconstruction by using alternative materials to titanium for cranial implants and analyzing their mechanical performance using the FEM. A 3D model of a skull with a cranial implant was created, and stress and strain studies were conducted on the skull and brain under different loading scenarios using materials like alumina, zirconia, hydroxyapatite, zirconia-reinforced PMMA, and PMMA. The following conclusions were drawn within the limitations of the study:

1. Stiff implants (titanium, alumina, zirconia, and PMMA + ZR) did not exceed their yield strengths, suggesting no failure may occur.
2. Soft implants (HA and PMMA) exceeded their strengths and could fail under 2000 N force.
3. Stiff ceramic implants (alumina and zirconia) reduced stresses, strains, and deformations on the skull and brain compared to PMMA+ZR, hydroxyapatite, and PMMA implants.
4. Alumina implants (the highest stiffness) produced the lowest values of deformations, maximum and minimum principal stresses and strains on the skull and brain.
5. PMMA implant (the lowest stiffness) produced the highest values of deformations, maximum and minimum principal stresses and strains on the skull and brain.
6. Zirconia-reinforced PMMA reduced stresses and strains compared to PMMA implants.

Finally, zirconia and alumina ceramic implants could be used as alternatives to titanium in cranioplasty, providing protection to the brain and skull without risk of fracture. Soft implants like HA and PMMA were not recommended due to their risk of fracture and high stress on the skull.

## Data Availability

The data used to support the findings of this study are included in the article.
